# Determination of the Stage Composition of *Plasmodium* Infections from Bulk Gene Expression Data

**DOI:** 10.1128/msystems.00258-22

**Published:** 2022-07-05

**Authors:** Kieran Tebben, Aliou Dia, David Serre

**Affiliations:** a Institute for Genome Sciences, University of Maryland School of Medicine, Baltimore, Maryland, USA; b Department of Microbiology and Immunology, University of Maryland School of Medicine, Baltimore, Maryland, USA; UCSF

**Keywords:** RNA-seq, gene expression deconvolution, *Plasmodium*, transcriptomics

## Abstract

Malaria symptoms are caused by the development of the parasites within the blood of an infected host. Bulk RNA sequencing (RNA-seq) of infected blood can reveal interactions between parasites and the host immune system during an infection, but because multiple developmental stages with distinct transcriptional profiles are concurrently present in infected blood, it is necessary to correct such analyses for differences in cell composition among samples. Gene expression deconvolution is a statistical approach that has been developed for inferring the cell composition of complex tissues characterized by bulk RNA-seq using gene expression profiles from reference cell types. Here, we describe the evaluation of a species-agnostic reference data set that can be used for efficient and accurate gene expression deconvolution of bulk RNA-seq data generated from any *Plasmodium* species and for correct gene expression analyses for biases caused by differences in stage composition among samples.

**IMPORTANCE** Differences in cell type proportions among samples can introduce artifacts in gene expression analyses and mask genuine differences in gene regulation. Gene expression deconvolution allows estimation of the proportion of each cell type present in one sample directly from bulk RNA sequencing data, but this approach requires a reference data set with the signature profile of each cell type. Here, we evaluate the suitability of a rodent malaria parasite gene expression data set for estimating the proportions of each parasite developmental stage present in bulk RNA sequencing data generated from blood-stage infections with the human parasites Plasmodium falciparum and Plasmodium vivax. These analyses provide a species-agnostic approach for reliably estimating stage proportions in infected human blood and correcting subsequent gene expression analyses for these variations.

## INTRODUCTION

Despite decades of progress toward elimination, *Plasmodium* parasites, the causative agents of malaria, remain endemic in 85 countries (1). In 2020, these parasites caused 241 million infections and over 600,000 deaths (most in children younger than 5 years), a significant increase compared to previous years (1). This reversal of progress, possibly due to emerging resistance to the most effective therapies (2), highlights the need for continued research into these medically important parasites.

At least five species of *Plasmodium* parasites cause human infections: P. falciparum, P. vivax, P. malariae, P. ovale, and P. knowlesi (3). P. falciparum causes the vast majority of malaria cases in Africa, while P. vivax causes most cases in South America and Southeast Asia (1). All these *Plasmodium* species share a complex life cycle that includes several distinct developmental stages across two hosts: humans and *Anophelines* mosquitoes. However, all malaria symptoms are caused by the intraerythrocytic development cycle (IDC) (4) of the parasites in the blood. After maturing in the liver, merozoites are released into the bloodstream, invade circulating erythrocytes, and mature into rings, trophozoites, and schizonts (4). Blood schizonts eventually release new merozoites that infect new red blood cells (RBCs) and continue the IDC (4). Asexual replication of parasites during the IDC occurs every 24 to 72 h (depending on the *Plasmodium* species) and increases the parasite load by 10- to 30-fold each cycle (4). A fraction of asexual parasites also differentiate into sexual stages (i.e., male and female gametocytes) that, once they are taken up by a mosquito during a blood meal, can continue their development and support malaria transmission (4).

Given the central role that blood-stage parasites play in disease severity, immune evasion, and transmission, analyses of parasite gene expression from patients’ blood would best allow us to understand how these parasites are regulated and interact with their host. However, relatively few studies have characterized the parasite gene expression profiles directly from patients (5–7). The main challenge of analyzing patient blood is the concurrent presence of multiple parasite stages in the blood (8), each with their own gene expression profiles (9, 10). This simultaneous presence of multiple stages *in vivo* is observed for all *Plasmodium* species, although to a lesser extent with P. falciparum due to the sequestration of mature stages (11, 12). Importantly, the proportions of the different blood stages vary with time and among patients, which can confound gene expression analyses: without controlling for the stage composition, differences in gene expression observed between samples might reflect differences in composition rather than transcriptional differences. To circumvent this limitation, several studies have used *in vitro* or short-term *ex vivo* cultures to synchronize parasites. However, these studies are limited to specific *Plasmodium* species (e.g., P. falciparum [13, 14] or P. knowlesi [15]), suffer from relatively low throughput, and might not fully recapitulate the *in vivo* parasite transcriptional profiles (15–18). A recent and appealing alternative is to use single-cell RNA sequencing (scRNA-seq) to characterize the heterogenous parasite populations present in one sample (13, 19). Unfortunately, these studies (i) are difficult to implement with patient samples due to the need for intact cells (which complicates storage), (ii) are expensive, and (iii) only provide a superficial characterization of the transcriptomes (since only the most expressed genes are robustly characterized). In this context, several approaches (20–22) have been used to estimate the proportion of the different stages present in a sample characterized by bulk RNA sequencing. Many methods rely on a prespecified set of marker genes for each cell type (i.e., parasite developmental stage) (23), which is problematic, as few *Plasmodium* genes are consistently expressed at a single stage (19). One alternative approach that is used extensively to differentiate cell types in complex human tissues is to leverage the information for hundreds of genes by using gene expression deconvolution (23, 24). We used this approach previously to analyze *Plasmodium*-infected blood samples (5, 7), but the robustness and accuracy of this method need to be rigorously evaluated.

CIBERSORTx (25) is a commonly used gene deconvolution software that first generates a “signature matrix” (i.e., a signature gene expression profile for each cell type) from a scRNA-seq reference data set and then uses this matrix to estimate the proportion of each of the cell types present in the sample characterized by bulk RNA-seq (25). While several scRNA-seq data sets have been generated for several *Plasmodium* species (13, 19, 26–28), the most comprehensive data set to date that captures parasites throughout the full IDC as well as male and female gametocytes comes from Plasmodium berghei (13), a rodent parasite distantly related to human malaria parasites. The ability to detect all blood stages, including gametocytes, in patient samples is essential to fully account for their presence and correct gene expression analyses for their variations among samples. Importantly, cell types that are present in bulk data but not included in the reference data set are redistributed across all other cell populations during deconvolution (23), which leads to biased estimates of all other cell types in the mixture (23).

Here, we evaluated the performance and limitations of different *Plasmodium* scRNA-seq data sets for gene expression deconvolution of both *in vivo* and *in vitro* samples. We provide a species-agnostic signature matrix to reliably analyze bulk RNA-sequencing data from any human *Plasmodium* parasites, allowing correction of gene expression analyses for parasite developmental stages present during an infection.

## RESULTS

### Single-cell RNA-seq data can reliably deconvolute bulk RNA-seq data from the same species.

**FIG 1:**
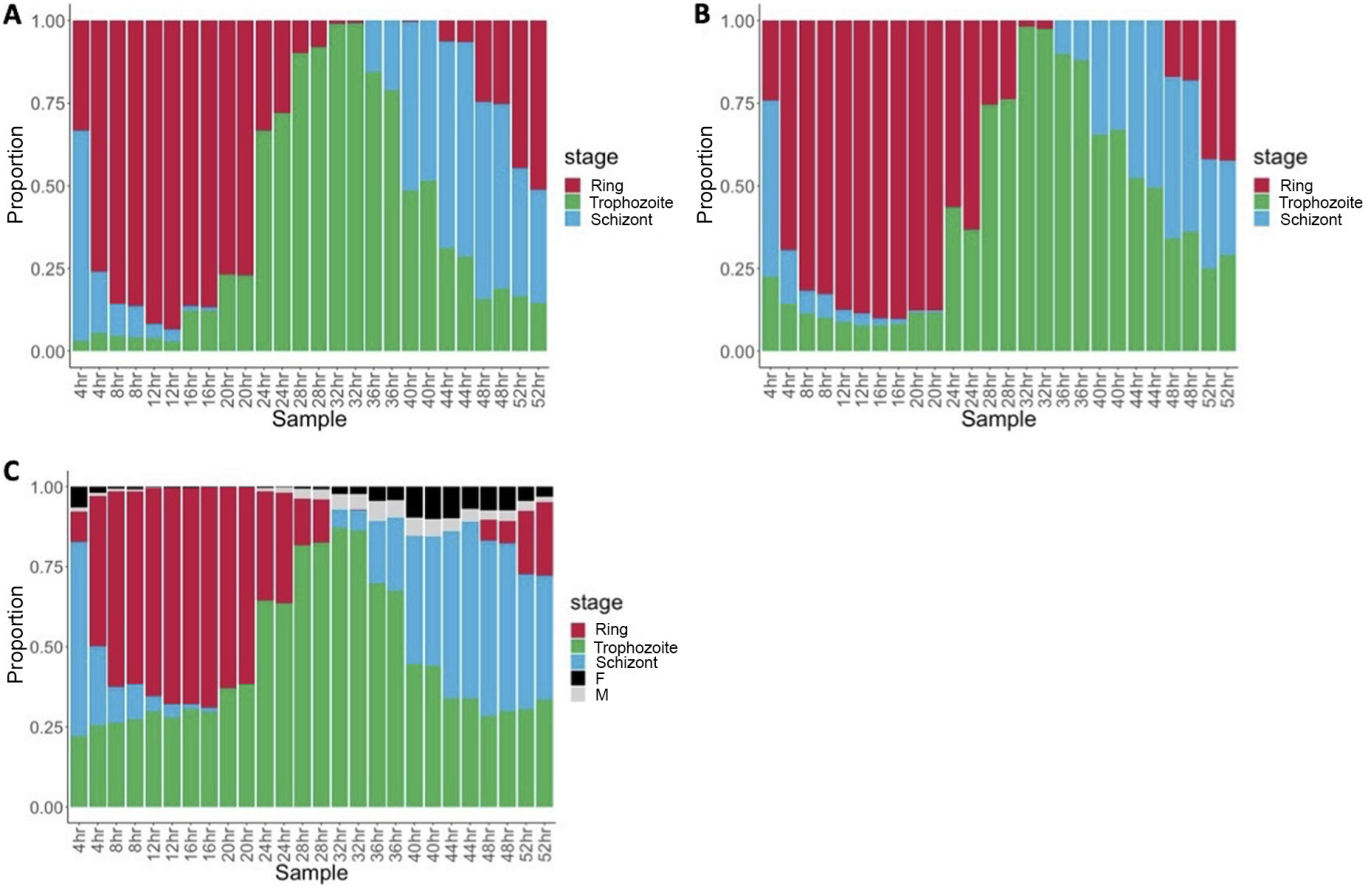
scRNA-seq data can reliably deconvolute bulk RNA-seq data. Each vertical bar represents one RNA-seq experiment generated from a synchronized P. falciparum *in vitro* culture (organized chronologically along the *x* axis) and is colored according to the proportion of transcripts derived from Ring (red), Trophozoite (green), Schizont (blue), Female gametocytes (black), and Male gametocytes (gray), as determined by CIBERSORTx. (A) Deconvolution of P. falciparum bulk RNA-seq data using all 4,923 P. falciparum genes of the scRNA-seq data to generate the signature matrix. (B) Deconvolution of the same P. falciparum bulk data using only the 3,391 P. falciparum genes with 1:1:1 orthologs to generate the signature matrix. (C) Deconvolution of the same P. falciparum bulk data using the 3,509 P. berghei genes with 1:1:1 orthologs from a scRNA-seq data set to generate the signature matrix.

We first tested whether scRNA-seq data sets could be used to deconvolute *Plasmodium* bulk gene expression data from the same species. We used CIBERSORTx (25) to generate signature profiles from a P. falciparum scRNA-seq expression data set (13) that encompassed three asexual stages—rings, trophozoites and schizonts—using all P. falciparum genes expressed (see Materials and Methods for details). We then used this signature matrix to deconvolute bulk RNA-seq data generated from a tightly synchronized P. falciparum culture sampled at 4-h intervals (13 time points, each in duplicate) (14). Overall, the gene expression deconvolution performed well, recapitulating the gradual progression of these samples through the IDC (Fig. 1A). CIBERSORTx (25) determined that the majority of the mRNAs from early samples (0 to 12 h postsynchronization) were derived from rings, while transcripts from trophozoites were most abundant in intermediate samples (24 to 32 h postsynchronization). We expected schizonts to dominate late cultures but these samples, while they did show the highest proportions of schizonts, seemed more heterogenous than those from earlier time points, possibly reflecting a gradual loss of synchronicity of the parasites as the cultures progressed.

The next step toward obtaining a universal signature matrix to deconvolute mixtures of blood-stage parasites from any *Plasmodium* species was to test whether reducing the number of genes used to generate the signature matrix lowered the efficiency and resolution of the gene expression deconvolution. We therefore reiterated the gene expression deconvolution of the same *in vitro* cultures but used only the 3,541 genes with 1:1:1 orthologs in the P. berghei, P. falciparum, and P. vivax genomes (instead of all 4,923 P. falciparum genes) to generate the signature matrix. Reducing the number of genes only minimally impacted the deconvolution results and yielded very comparable results (Fig. 1B).

### Single-cell RNA-seq data from one *Plasmodium* species can reliably deconvolute RNA-seq data from another species.

We next tested whether scRNA-seq data generated from the rodent parasite P. berghei could be used to deconvolute P. falciparum bulk data. We selected P. berghei as a possible reference here since the available scRNA-seq for this species included all asexual blood-stage parasites (as in the P. falciparum data above), as well as male and female gametocytes, which we expect to be present in at least some of the infected patient blood samples. We used the P. berghei scRNA-seq data set (13), including only genes with 1:1:1 orthologs in P. berghei, P. falciparum, and P. vivax, to generate a signature matrix and deconvolute the bulk RNA-seq data described above. Overall, the proportions of each developmental stage estimated in each sample were very similar to the results obtained using P. falciparum as reference, suggesting that using only genes conserved across *Plasmodium* species performed quite efficiently (although the gene expression deconvolution seemed to systematically infer more trophozoites in all culture samples) (Fig. 1C). Importantly, inclusion of gametocytes in the signature matrix did not lead to inferences of many gametocytes in the culture samples; we did not expect gametocytes to be present in these culture samples and, indeed, less than 10% gametocytes were inferred by deconvolution (Fig. 1C).

**FIG 2:**
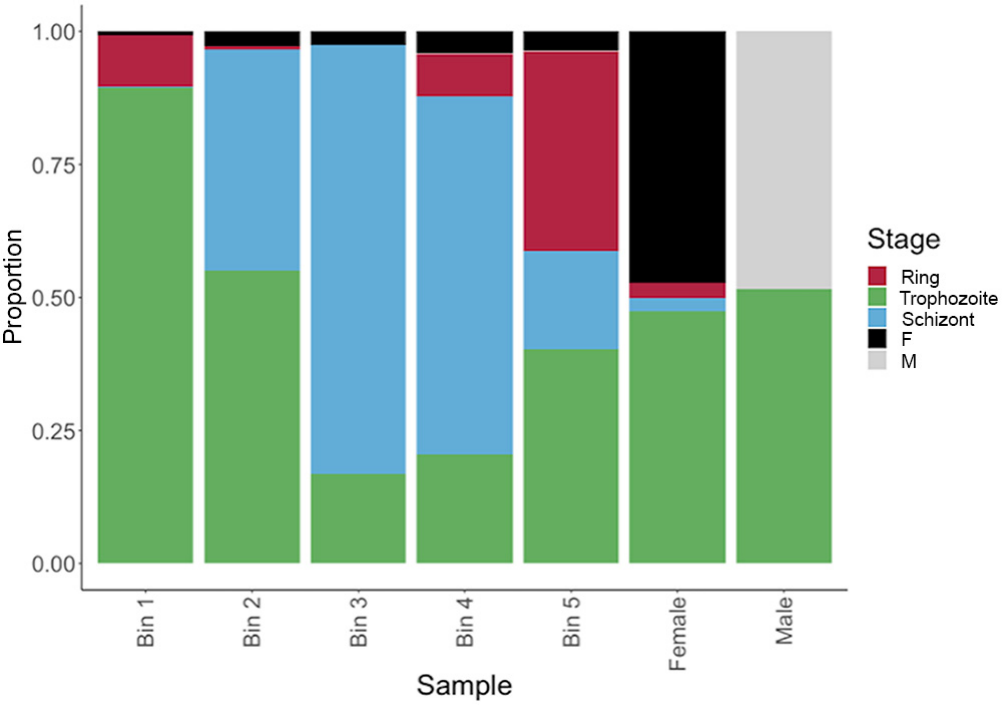
A species-agnostic reference matrix can be used to deconvolute P. vivax bulk mixtures, as shown by this deconvolution of P. vivax mock bulk data with a signature matrix generated from P. berghei scRNA-seq single-cell expression profiles. Cells were binned into equal bins by pseudotime to represent progression from early trophozoites (bin 1) to late schizonts (bin 5) along the *x* axis.

We also evaluated how this species-agnostic signature matrix would perform on P. vivax blood-stage parasites. Since we lacked bulk RNA-seq data from synchronized P. vivax parasites, we generated mock RNA-seq data by aggregating scRNA-seq data from individual parasites at the same stages (19) (see Materials and Methods). The P. vivax scRNA-seq data set did not include ring-stage parasites (due to limitations of the enrichment method used before library preparation [19]), and we therefore generated five data sets representing P. vivax parasites across the IDC from early trophozoites (bin 1) to late schizonts (bin 5). In addition, we included two mock populations made of male or female gametocytes. The proportions inferred by gene expression deconvolution for each mock data point approximately matched the expected proportions (Fig. 2), although with an apparent systematic overestimation of trophozoites, similarly to Fig. 1. Interestingly, despite the use of a signature derived from P. berghei gametocytes, P. vivax gametocytes of each sex were correctly identified (despite a high “misassignment” to trophozoites; see below).

### Gene expression deconvolution provides a robust but relative assessment of stage composition across samples.

**FIG 3:**
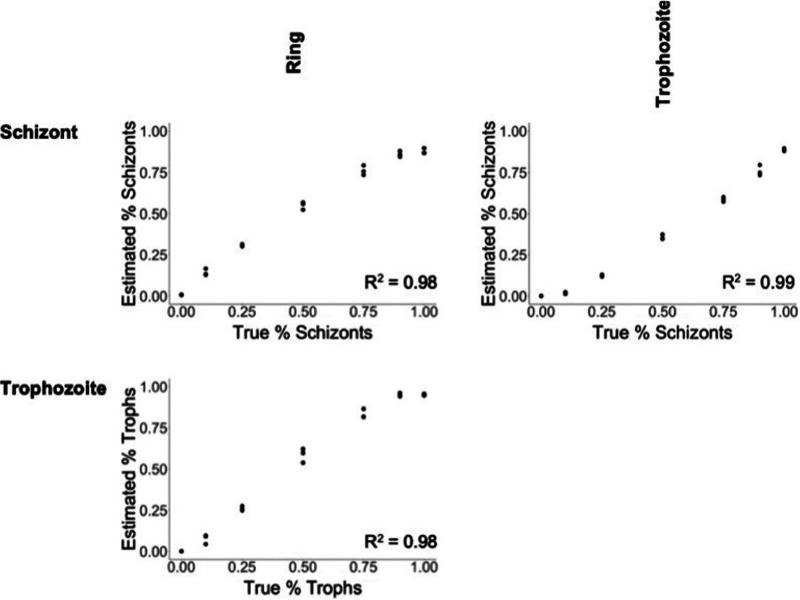
Accuracy of gene expression deconvolution using a species-agnostic signature matrix. Each plot displays the estimated proportion of a specific P. berghei stage each cell type (*y* axis) in simulated mixtures compared to the true proportions (*x* axis).

We then assessed the accuracy of the gene expression deconvolution by analyzing mock bulk gene expression data generated by mixing P. berghei parasites from different developmental stages. We randomly sampled 100 cells from selected stages from the P. berghei scRNA-seq data set (13) to obtain mixed populations of two stages in different proportions. We then compared the estimated proportions obtained after deconvolution with the true proportions of mixed cells. In all mock mixtures, the estimated proportion of each stage increased linearly with the true proportion of that stage, indicating that deconvolution is highly accurate (Fig. 3).

When this method was applied to mock mixtures of the P. falciparum scRNA-seq data, we still observed a monotonic increase in estimated percentage of each stage as the actual percentage increased, reinforcing that our species-agnostic signature matrix is efficient across species (see Fig. S1 in the supplemental material). However, the proportion of schizonts was systematically underestimated in all mock mixtures. This could be due to inclusion, in the mock mixtures, of parasites labeled as schizonts in the scRNA-seq data but actually representing earlier parasites than the P. berghei schizonts and were therefore assigned by deconvolution into a different group.

10.1128/msystems.00258-22.1 FIG S1 Accuracy of gene expression deconvolution using a species-agnostic signature matrix. Each plot displays the estimated proportion of a specific P. falciparum stage of each cell type (*y* axis) in simulated mixtures compared to the true proportions (*x* axis). Note that the maximum proportion estimated is different from the “true” one and likely reflects mislabeling of some of the cells used for generating the mock mixtures (see main text for details). Download FIG S1, JPG file, 0.05 MB. Copyright © 2022 Tebben et al. 2022 Tebben et al. https://creativecommons.org/licenses/by/4.0/ This content is distributed under the terms of the Creative Commons Attribution 4.0 International license.

## DISCUSSION

Here, we evaluated the performance of CIBERSORTx, a gene expression deconvolution program (29), for estimating the stage composition of *Plasmodium* blood infections characterized by bulk RNA-seq. This approach has been used previously for analyzing blood samples from malaria patients (5, 7) but without comprehensive benchmarking of its accuracy and resolution. We showed here that we can use this approach to leverage the transcriptional profiles determined from P. berghei parasites by scRNA-seq data to reliably estimate the proportions of the blood stages of human *Plasmodium* infections characterized by bulk RNA-seq.

One key feature of gene expression deconvolution is that it relies on hundreds of genes to determine the signature profiles of the reference populations or stages (see below). We showed that CIBERSORTx performance was not dramatically reduced when we used only the subset of genes conserved across *Plasmodium* species. We then demonstrated that we could use scRNA-seq data from P. berghei, a distantly related rodent malaria parasite, to efficiently deconvolute data generated for the human parasites P. falciparum and P. vivax. This result, which derives from the overall conservation of the regulation of *Plasmodium* gene expression throughout their life cycle (30, 31), is critical, as P. berghei is currently the species most comprehensively characterized and for which scRNA-seq data are available for all asexual and sexual blood stages. In addition, the P. berghei scRNA-seq data were generated from short-term *ex vivo* cultures (13) and might therefore better recapitulate patient infections than data generated from parasites cultivated *in vitro* (15, 32).

The stage proportions estimated by gene expression deconvolution should be interpreted with caution. First, it is likely that this approach will systematically underestimate specific stages: gene expression deconvolution estimates the proportion of the transcripts derived from each stage but, since some *Plasmodium* stages are much more transcriptionally active than others (33), the gene expression deconvolution might not match microscopy results 1 to 1. For example, while P. falciparum ring-stage parasites are expected to predominate in patients due to sequestration (11, 12), their relatively low transcriptional activity, compared to schizonts and trophozoites (33), could lead to an underestimation of their abundance (i.e., ring transcripts will account for a lower fraction of all mRNAs than expected based on cell numbers). Second, the results are dependent on the “reference” populations used. While development of blood stage along the IDC and the changes in gene expression are continuous (13, 19), we used discrete categories to assign parasites into stages and, consequently, samples containing late trophozoites might be represented as a mixture of trophozoites and schizonts. This could complicate interpretations of the estimated stage composition and comparisons between data sets deconvoluted with different references. However, this limitation does not hamper the correction of gene expression data from patient samples for stage differences, since the same biases will affect all samples similarly. The linear correlation between the true and estimated proportions of variable mixtures (Fig. 3) is essential in this regard, as it ensures that correction for stage differences between samples will rely on, if not absolute estimates, at least estimates proportional to the true proportions and therefore correct appropriately for variations among samples.

Several approaches have been previously applied to correct *Plasmodium* gene expression analyses for differences in stage composition among samples. Several studies have measured the overall correlation between the profiles of bulk samples with synchronized cultures at different time points to cluster samples according to their overall stage distribution (20, 22). While this approach allows identification of outlier samples with drastically different distributions, it may fail to fully account for more subtle differences in stage compositions. Alternatively, some studies have used carefully selected “marker” genes specific to each stage (21) to estimate the relative proportions of different stages. While conceptually similar to the gene expression deconvolution method implemented in CIBERSORTx, this approach relies on a handful of selected genes, and any difference in the regulation of these marker genes will lead to biases in the estimation of the stage composition. This shortcoming highlights the main advantage of CIBERSORTx: because it uses the combination of hundreds of genes as “signatures,” the deconvolution is robust to changes in the regulation of specific genes.

By robustly estimating relative proportions of each sexual and asexual stage present in an infection characterized by bulk RNA-seq, gene expression deconvolution alleviates the main limitation of using infected blood samples, without culture, for *Plasmodium* gene expression analyses: without rigorously estimating the stage composition of each sample, it is difficult to determine whether gene expression differences between samples are due to (i) genuine differences in gene expression between parasites or (ii) differences in the developmental stages present in each sample. Due to sequestration (11, 12), most P. falciparum parasites present in the circulation tend to be early-stage parasites (i.e., rings). However, even a minority of late-stage parasites might dramatically affect the overall RNA-seq profiles, since those parasites can disproportionally contribute to the mRNA populations due to their higher transcriptional activity (33). In addition, P. falciparum infections can vary extensively in the proportion of sexual parasites present at a given time (34). In non-falciparum infections, this stage heterogeneity is magnified by the lack of sequestration, which enables all parasite stages to be simultaneously present in the bloodstream and, in the case of P. vivax, the earlier appearance of gametocytes in an infection (34). Our analyses provide a species-agnostic solution to robustly address this heterogeneity. Using a comprehensive P. berghei scRNA-seq data set, including the detailed descriptions of the transcriptional profiles of sexual and asexual blood-stage parasites, CIBERSORTx enables rigorous estimation of the stage compositions of bulk samples which can then be used as covariates in testing for differential expression between groups. This simple method will allow analysis of large numbers of blood samples, without the need to culture the parasites before profiling (which is both time- and resource-consuming), and use of standard bulk RNA-seq methods, which are much cheaper than scRNA-seq and provide a deeper characterization of the transcriptome. Overall, the method described in this study will provide a solid foundation to implement *Plasmodium* gene expression analyses for patient infections and to better understand the role of these parasites in disease etiology and severity (e.g., by comparing symptomatic versus asymptomatic infections, or uncomplicated malaria cases to cases of severe malaria), or to examine the molecular mechanisms underlying immune evasion.

## MATERIALS AND METHODS

### scRNA-seq data sets and preprocessing.

We used two publicly available 10× Genomics single-cell RNA-seq data sets from Howick et al. (13): one characterizing stages of the intraerythrocytic developmental cycle (IDC) of P. falciparum and one for P. berghei that included gametocytes in addition to asexual parasites. Raw count tables were downloaded for 6,737 cells representing the P. falciparum IDC (stages: ring, early trophozoite, late trophozoite, and schizont) and 4,884 cells representing the P. berghei blood stages (stages: early ring, late ring, early trophozoite, mid-trophozoite, late trophozoite, early schizont, mid-schizont, late schizont, male gametocyte, and female gametocyte). To ensure that enough cells were included to accurately recapitulate the gene expression profile of a specific stage, we pooled cells at slightly different time points within broadly defined developmental stages. For the P. falciparum data set, cells labeled as early and late trophozoites were grouped together as “trophozoites” (while parasites labeled as rings and schizonts were unchanged). For the P. berghei data set, cells labeled as early and late rings were pooled together as “rings”; early, mid-, and late trophozoites were grouped into “trophozoites”; and early, mid-, and late schizonts were grouped into “schizonts.”

We also used a 10× Genomics scRNA-seq data set generated from blood-stage P. vivax parasites (19) (without ring parasites, since these were lost during enrichment). A raw count table was available for 9,215 blood-stage parasites, including male and female gametocytes. Individual stage labels were not available for IDC stages, so cells were binned by pseudotime into five bins of equal length, representing progression from early trophozoites to late schizonts. Male and female gametocytes were individually labeled.

### Bulk RNA-seq data sets and preprocessing.

To evaluate the efficiency of deconvolution, we used one bulk RNA-seq data set (14) and included 13 time points along a highly synchronized P. falciparum 3D7 culture (in 4-h increments, with duplicates of each time point, for 56 h total [35]). Cultures of mostly rings (by microscopy) were synchronized with sorbitol (35). Two additional sorbitol synchronizations at 10 h and 28 h after initial synchronization ensured tightly synchronized cultures (35). Each sample was labeled with its corresponding time point (in hours postsynchronization) and stage (ring, trophozoite, or schizont), confirmed by microscopy. Read counts were collapsed by gene by summing the counts determined from each isoform.

### Orthologous genes across three *Plasmodium* species.

A table of orthologous genes in P. falciparum, P. berghei, and P. vivax was obtained from PlasmoDB (36). Briefly, we downloaded a table of all P. falciparum 3D7 genes and their orthologs in the P. vivax P01 and P. berghei ANKA genomes. We then removed all genes with no or multiple orthologs in any of the three species, retaining a total of 3,541 1:1:1 orthologous genes.

### Generation of signature matrices.

We generated and evaluated three signature matrices from the scRNA-seq data: (i) P. falciparum scRNA-seq including all 4,923 genes in the data set; (ii) a P. falciparum scRNA-seq subset to include only genes with 1:1:1 orthologs; and (iii) a P. berghei scRNA-seq data set subset to include only 1:1:1 orthologs (with the P. berghei gene names replaced by orthologous P. falciparum or P. vivax gene names).

### Mock mixture data.

We generated mock RNA-seq data by “mixing” 500 parasites from the P. falciparum or P. berghei scRNA-seq data set using varying proportions of each stage. Randomly sampled cells from the selected stages were collapsed by sum to generate a single mixture data set. We generated these data sets by simply summing the actual scRNA-seq read counts per gene.

### CIBERSORTx.

Each reference data set (described above) was uploaded to CIBERSORTx (25) and used to generate a signature matrix defining the gene expression profiles of each developmental stage in the data set. The minimum expression threshold was reduced from 0.75 (default) to 0 to reduce data sparsity resulting from the smaller number of genes captured in 10× data sets and ensure enough genes were included to generate a reliable signature matrix. All other parameters were used with default settings.

CIBERSORTx (25) was then used to estimate proportions of each cell type in the bulk data sets described above. Batch correction was enabled in S-mode to correct for sequencing differences between single-cell and bulk data sets. Quantile normalization was disabled. Default settings were used for all other parameters, with 500 permutations for analysis.

### Code availability.

All programs, versions, and parameters used in this study are described in Materials and Methods. Custom codes and gene lists are available at https://github.com/tebbenk/GED.
