# Supplementary figures and images for: Determination of the Stage Composition of Plasmodium Infections from Bulk Gene Expression Data
Source: mSystems. 2022 Jul 5;7(4):e00258-22. doi: 10.1128/msystems.00258-22 (PMC9426464; doi:10.1128/msystems.00258-22)

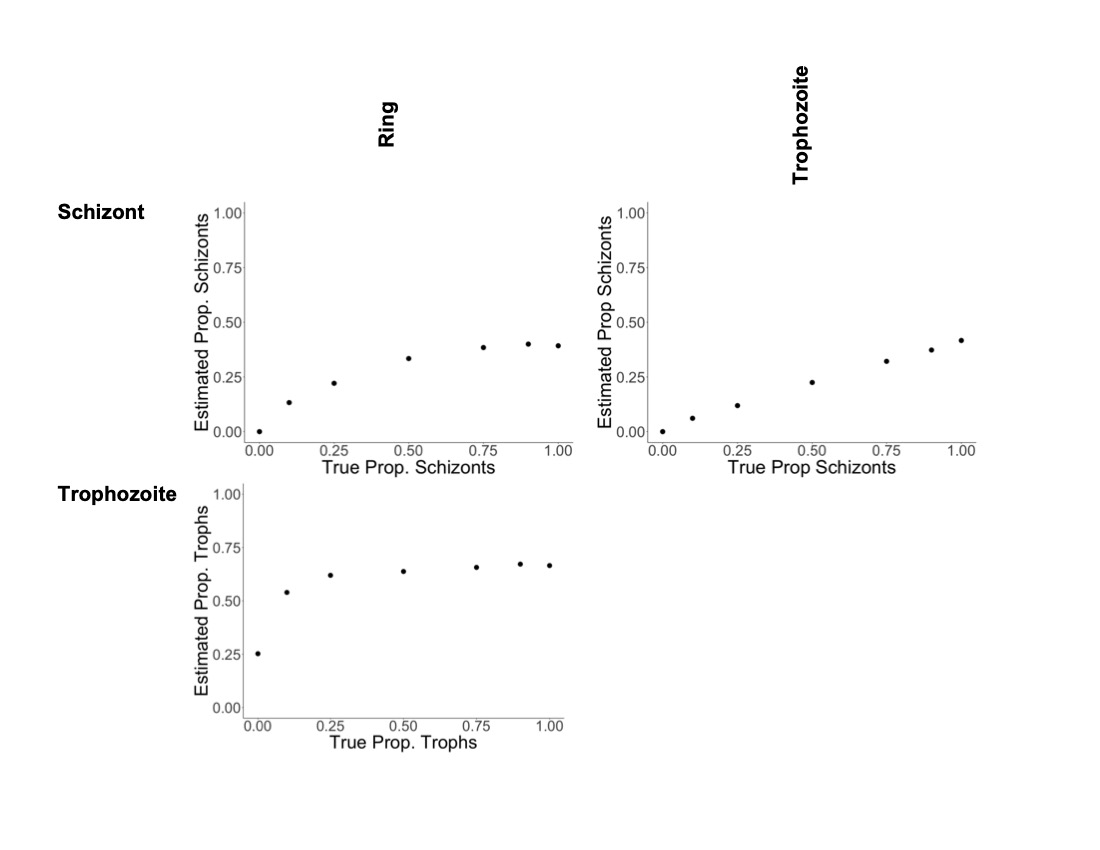

Supplement: FIG S1 [file msystems.00258-22-s0001.jpg]
